# Novel Biphasic Role of Resolvin D1 on Expression of Cyclooxygenase-2 in Lipopolysaccharide-Stimulated Lung Fibroblasts Is Partly through PI3K/AKT and ERK2 Pathways

**DOI:** 10.1155/2013/964012

**Published:** 2013-09-23

**Authors:** Derong Wu, Shengxing Zheng, Wenjuan Li, Li Yang, Yongjian Liu, Xia Zheng, Yi Yang, Liangmin Yang, Qian Wang, Fang Gao Smith, Shengwei Jin

**Affiliations:** ^1^Department of Anesthesia and Critical Care, Second Affiliated Hospital of Wenzhou Medical University, 109 Xueyuan Road, Wenzhou, Zhejiang Province 325027, China; ^2^Academic Department of Anesthesia, Critical Care, Pain and Resuscitation, Birmingham Heartlands Hospital, Heart of England NHS Foundation Trust, Birmingham B9 5SS, UK

## Abstract

Fibroblasts, far frombeing merely bystander cells, are known to play a specific role in inflammation resolution after an acute injury. As the endogenous “braking signal,” resolvins possess potent anti-inflammatory and proresolution actions. We demonstrated that the expression of COX-2 protein was significantly peaked initially at 6 hours but then also at 48 hours after LPS stimulation in lung fibroblasts. PGE_2_ levels also peaked at 6 hours, and PGD_2_ levels were increased and peaked at 48 hours. However, no significant change in the protein expression of COX-1 was observed after treatment with LPS in lung fibroblasts. Exogenous resolvin D1 inhibited the first peak of COX-2 expression as well as the production of PGE_2_ induced by LPS. In contrast, exogenous resolvin D1 increased the second peak of COX-2 expression as well as the production of PGD_2_ induced by LPS. In addition, resolvin D1 inhibited COX-2 expression at 6 hours, which was partly through PI3K/AKT and ERK2 signalling pathways.

## 1. Introduction

The main pathophysiology of acute respiratory distress syndrome (ARDS) consists of overlapping acute “inflammatory” and delayed “repair/fibrotic” phases [1, 2]. However, mechanisms regulating the resolution of ARDS are poorly understood. Fibroblasts, which are far from being merely bystander cells, are important to host defence [3]. A study reported that fibroblasts exhibit a persistent activated phenotype with enhanced migratory and collagen I production capacities in comparison with control fibroblasts [4]. During inflammation, fibroblasts become activated and produce inflammatory mediators, including interleukin-8 (IL-8), monocyte chemoattractant protein-1, and express cyclooxygenase-2 (COX-2), with the resultant release of proinflammatory prostaglandins (PGs) such as prostaglandinE_2_ (PGE_2_) [5, 6]. Moreover, fibroblasts may also promote early fibroproliferative response to ARDS correlating with poor outcomes [7, 8].

However, fibroblasts are known to play a specific role in inflammation resolution after an acute injury [9]. Numerous studies suggest that fibroblasts are key cells to secrete factors (such as basic fibroblast growth factor, bFGF, and keratinocyte growth factor, KGF) that aim to upregulate repair of the damaged alveolar blood/air barrier of the lung [10–12]. In addition, the presence of alveolar fibroblasts is associated with a reduction of ventilation duration and with a decrease of inflammatory markers and could reflect an adapted repair process contributing to the resolution of acute lung injury [4].

Cyclooxygenase is a resourceful mode of formation of specific autacoids that regulate the extent and pace of the inflammatory response. Cyclooxygenase is expressed in cells in three distinct isoforms, cyclooxygenase-1 (COX-1), cyclooxygenase-2 (COX-2), and cyclooxygenase-3 (COX-3) [13]. COX-1 is expressed in most mammalian cells under physiological conditions, whereas COX-2 expression is inducible by a variety of extracellular and intracellular stimuli, including LPS, TNF-*α*, or IL-1*β* [13, 14], and COX-3, which is a variant of COX-1 and detected mainly in the central nervous system [13]. Prostaglandins, oxygenated metabolites of arachidonic acid, are important mediators and modulators of the inflammatory response to infection. Prostaglandins (PGs) have received much attention because of their seemingly dichotomous nature. ProstaglandinE_2_ (PGE_2_), the main prostaglandin produced during inflammatory response, participates in initiation of inflammation [15]. Previous studies suggest that prostaglandinD_2_ (PGD_2_), as a proresolution mediator, also actively leads to the resolution of tissue injury and inflammation [16].

The resolvins (resolution phase interaction products) are a family of lipid mediators derived from both eicosapentaenoic acid (EPA) and docosahexaenoic acid (DHA); they were termed as E series resolvins (RvE) and D series resolvins (RvD), respectively [17]. Evidence indicates that resolvins possess potent anti-inflammatory and proresolution actions. Resolvin D1 has recently been identified to inhibit PMN infiltration and transmigration [18–20], reduce interstitial fibrosis [21], regulate cytokines to sites of inflammation [22, 23], and protect after ischemia-reperfusion second organ injury [21, 24] and LPS-induced acute lung injury [25]. 

A recent study reported that COX-2 played a pivotal role in the resolution of acute lung injury and uncovered a new role for COX-2-derived lipid mediators in promoting resolution of acid-initiated experimental acute lung injury [26]. In response to acid injury, epithelial cells rapidly increased COX-2 expression and PGE_2_ production [27]. The COX-2 enzyme has also been identified as an important mediator of pulmonary fibrosis, with COX-2^−/−^ mice having increased fibrotic lung responses [28]. More recently, resolvin D1 markedly reduced the expression of COX-2 on LPS-induced acute lung injury in mice [25], and fibroblasts are capable of expressing COX-2 and producing PGE_2_ directly stimulated by LPS [5, 6]. However, the time course of COXs (COX-1 and COX-2) expression in lung fibroblasts stimulated by LPS and the effect of resolvin D1 on expression of COXs (COX-1 and COX-2) and production of PGE_2_ and PGD_2_ remain unclear. 

In this study, we examined the expression of COXs (COX-1 and COX-2) and the production of PGE_2_ and PGD_2_ in lung fibroblasts after LPS challenge. Additionally, we also investigated the effect of resolvin D1 on the expression of COXs (COX-1 and COX-2) and the production of PGE_2_ and PGD_2_. Finally, we further revealed its underlying mechanism. We present evidence for a novel biphasic role of resolvin D1 on the expression of COX-2 and the production of PGE_2_ and PGD_2_ in LPS-stimulated lung fibroblasts and highlight a new sight for the role of fibroblasts in acute respiratory distress syndrome. 

## 2. Material and Methods

### 2.1. Materials

Cell culture media (F-12K, DMEM), FBS, Trypsin EDTA were purchased from Gibco company (NY, USA). Penicillin and streptomycin in saline citrate buffer were from Invitrogen company (CA, USA). Lipopolysaccharide (LPS) UO126 and LY294002 were purchased from Sigma company (Santa Clara, USA). Antibodies against COX-1 and COX-2 were purchased from Abcam company (Cambridge, UK). Rabbit anti-rat threonine/tyrosine dephosphorylated ERK1/2 and serine phosphorylated AKT were purchased from Cell Signaling Technology company (Boston, USA). PGE_2_, PGD_2_, MCP-1, and IL-8 ELISA kits were purchased from R&D company. And resolvin D1 was Purchased from Cayman Chemical Company (MI, USA) and stored at −80°C until being diluted in the low-serum medium immediately before use. 

### 2.2. Cell Culture

The HFL-1 cell line (human fetal lung fibroblasts) was obtained from Shanghai Institute of Cell Biology, Chinese Academy of Sciences (Shanghai, China). HFL-1 were monolayers cultured in F-12K medium supplemented with 10% FBS. Cells were passaged every 2-3 days and used for experiments at passages 15–25.

Rat pulmonary fibroblasts were isolated from 2 days newborn Sprague-Dawley rats according to the manufacturer's protocol. Lung tissue was cut into <1 mm^3^ pieces and dissociated in Hanks buffered saline solution (HBSS) containing 0.25% trypsin at 37°C for 1.5 minutes. Trypsin was inhibited by DMEM with 15% FBS and dissociated tissue centrifuged at 1000 g for 5 minutes at 4°C. The dissociated tissue pieces were placed into a culture plate with DMEM containing 15% FBS and left to allow fibroblast outgrowth. After fibroblasts had grown out from the tissues, usually 1-2 days, the remaining tissue was removed by aspiration, and the cells were allowed to reach confluence. Confluent fibroblasts were then passaged with a split ratio of 1 : 2 by trypsin treatment and used for the experiments at passages 4–6.

For all experiments, cells (5 × 10^5^) were plated in 6-well plates and grown to 80% confluence. Then, cells were serum deprived for 24 hours in DMEM medium containing 0.5% FBS prior to the addition of LPS and/or resolvin D1. The cells were then incubated with LPS (1 *μ*g/mL) for 0, 6, 12, 24, 48, and 72 hours. 

### 2.3. Stimuli and Inhibitor

Fibroblasts were incubated in the low-serum medium containing 1 *μ*g/mL LPS for 6 hours or 48 hours in the presence or absence of 10, 50, or 100 nM of resolvin D1. Because resolvin D1 is dissolved in ethanol and the effect of ethanol should be taken into consideration, so we used ethanol as a control to evaluate the effect of resolvin D1 on expression of COXs and PGs induced by LPS. Inhibitors were used at the following concentrations according to manufacturers' instructions: LY294002, a selective PI3-K inhibitor at 30 *μ*M, was added to primary rat lung fibroblasts 30 minutes prior to every treatment. UO126, a selective ERK1/2 inhibitor, at 10 *μ*M, was added to primary rat lung fibroblasts 1 hour before every treatment. 

### 2.4. Western Blotting

Western blot analyses from cells homogenates were performed as described previously. After equal amounts of protein were loaded in each lane and separated by 10% SDS-PAGE, the proteins were transferred to polyvinylidene difluoride membranes (Millipore, Billerica MA01821). The membranes were blocked for 2 hours with 5% skimmed milk, which was also used as primary (1 : 1000) and secondary antibodies (1 : 1000) incubation buffer. The primary antibodies were incubated overnight at 4°C. Horseradish peroxidase-conjugated secondary antibodies were incubated for 2 hours at room temperature and imaged by the Image Quant LAS 4000 mini (GE). 

### 2.5. Measurement of PGE_**2**_, PGD_**2**_, IL-8, and MCP-1 Production

Fibroblast supernatants were collected following treatments, centrifuged (1500 g, 5 minutes), aliquoted, and stored at −80°C. PGE_2_, PGD_2_, IL-8, and MCP-1 protein expression was measured by ELISA according to the manufacturer's instructions (R&D systems). Assays were run in triplicate and repeated twice.

### 2.6. Statistic Analysis

Data are represented as mean ± SEM. All data were analyzed by Student's *t*-test or by one-way analysis of variance followed by Tukey's test for post hoc comparisons. *P* value < 0.05 was considered significant. Statistical analyses were performed using Prism 5.0 software (GraphPad Software, San Diego, CA).

## 3. Results

### 3.1. The Effect of LPS on COX-1, COX-2, PGE_**2**,_ PGD_**2**_, MCP-1, and IL-8 Expression in Lung Fibroblasts

**Figure 1:**
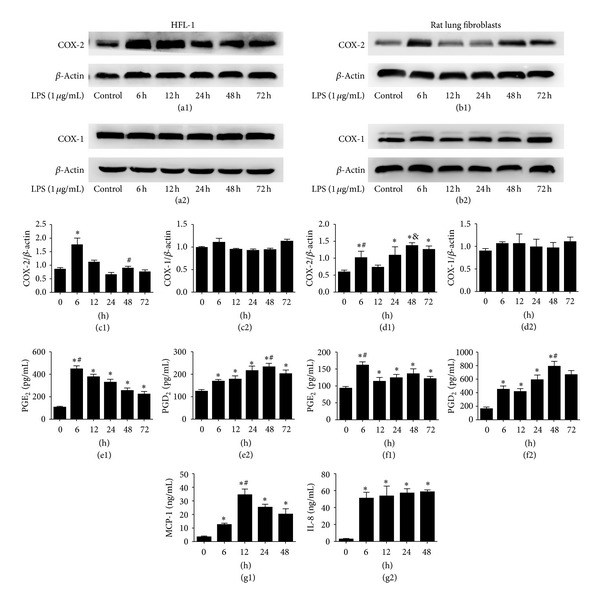
The effect of LPS on COX-1, COX-2, PGE_2,_ PGD_2_, MCP-1, and IL-8 expression in lung fibroblasts. ((a1), (a2), (c1), (c2)) HFL-1 cells were incubated with LPS (1 *μ*g/mL) for 6, 12, 24, 48, and 72 hours. The expression of COX-1 and COX-2 protein was assessed by western blot and analysed by densitometry compared to *β*-actin expression. The expression of COX-2 protein in HFL-1 cell line was increased and peaked both at 6 hours and 48 hours after LPS stimulation (^*∗*^ *P* < 0.05 versus control, 12, 24, 48, and 72 hours groups, ^#^ *P* < 0.05 versus 24 hours group). However, no significant change in the protein expression of COX-1 was observed after treatment with LPS in HFL-1 cell line. ((e1), (e2)) Supernatants were collected after LPS (1 *μ*g/mL) treatment for 6, 12, 24, 48, and 72 hours. PGE_2_ protein was measured by ELISA. Data are expressed as mean ± SE for each group (^*∗*^ *P* < 0.05 versus control, ^#^ *P* < 0.05 versus 12, 24, 48, and 72 hours groups). PGD_2_ protein was also assayed by ELISA. Data are expressed as mean ± SE for each group (^*∗*^ *P* < 0.05 versus control, ^#^ *P* < 0.05 versus 6, 12, 72 hours groups). ((b1), (b2), (d1), (d2), (f1), (f2)) We also dulicated our test in primary rat lung fibroblasts ((b1), (d1)), (^*∗*^ *P* < 0.05 versus control, ^#^ *P* < 0.05 versus 12 hour group, ^&^ *P* < 0.05 versus 6, 12, 24, and 72 hours groups). ((b2), (d2)) No significant change in the protein expression of COX-1 was observed after treatment with LPS in primary rat lung fibroblasts (^*∗*^ *P* < 0.05 versus control, ^#^ *P* < 0.05 versus 12, 24, 72 hours groups, (f1)) and (^*∗*^ *P* < 0.05 versus control, ^#^ *P* < 0.05 versus 6, 12, 24, 72 hours group, (f2)). All experiments were repeated in triplicate. ((g1), (g2)) Supernatants were collected after LPS (1 *μ*g/mL) treatment for 0, 6, 12, 24, and 48 hours in primary rat lung fibroblasts. MCP-1 protein was measured by ELISA. Data are expressed as mean ± SE for each group (^*∗*^ *P* < 0.05 versus control, ^#^ *P* < 0.05 versus 6, 24, 48 hours groups). IL-8 protein was also assayed by ELISA. Data are expressed as mean ± SE for each group (^*∗*^ *P* < 0.05 versus control).

To determine the dynamic expression of COXs (COX-1 and COX-2) in HFL-1 cell line and primary rat lung fibroblasts, cells were incubated with LPS (1 *μ*g/mL) for 0, 6, 12, 24, 48, and 72 hours. The expression of COX-2 protein in HFL-1 cell line was increased and peaked both at 6 hours and 48 hours after LPS stimulation (Figure 1(a1, c1)). The expression of COX-2 protein in primary rat lung fibroblasts was significantly increased and peaked initially at 6 hours after LPS stimulation, with maximal levels occurring at 48 hours (Figure 1(b1, d1)). In addition, PGE_2_ levels were peaked only at 6 hours both in HFL-1 cell line and primary rat lung fibroblasts. PGD_2_ levels increased and peaked at 48 hours in HFL-1 cell line as well as primary rat lung fibroblasts (Figure 1(e1, e2, f1, f2)). However, no significant change in the protein expression of COX-1 was observed after treatment with LPS both in HFL-1 cell line and primary rat lung fibroblasts (Figure 1(a2, b2, c2, d2)). During the inflammatory process, besides the expression of COX-2, the production of some proinflammatory chemokines, such as interleukin-8 (IL-8) and monocyte chemoattractant protein-1 (MCP-1) takes place. To determine the dynamic expression of MCP-1 and IL-8 in primary rat lung fibroblasts, cells were incubated with LPS (1 *μ*g/mL) for 0, 6, 12, 24, 48 hours. The expression of MCP-1 protein in primary rat lung fibroblasts was peaked only at 12 hours. In contrast, although IL-8 levels increased with LPS treatment, no significant change in the IL-8 protein was observed between 6, 12, 24, and 48 hours groups in primary rat lung fibroblasts (Figure 1(g1, g2)). 

### 3.2. The Effect of Resolvin D1 on Expression of COXs (COX-1 and COX-2) and PGE_**2**_ and PGD_**2**_ Production at 6 Hours in HFL-1 Cell Line and Primary Rat Lung Fibroblasts Stimulated with LPS

**Figure 2:**
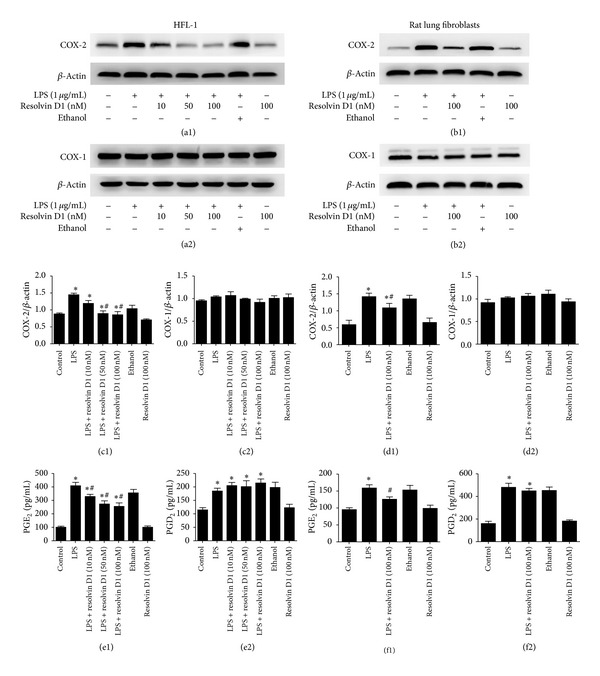
The effect of resolvin D1 on expression of COXs (COX-1 and COX-2) and PGE_2_ and PGD_2_ production at 6 hours in lung fibroblasts stimulated with LPS. ((a1), (a2), (c1), (c2)) HFL-1 cells were treated with resolvin D1 at 0, 10, 50, or 100 nM in the presence of LPS (1 *μ*g/mL) for 6 hours. COX-2 protein was detected by western blot (^*∗*^ *P* < 0.05 versus control, ^#^ *P* < 0.05 versus LPS, LPS + resolvin D1 (10 nM) groups). No significant change in the protein expression of COX-1 was observed after treatment with LPS in HFL-1 cells. ((e1), (e2)) Supernatants from HFL-1 cells treated with resolvin D1 at 0, 10, 50, or 100 nM in the presence of LPS (1 *μ*g/mL) for 6 hours were collected and PGE_2_ protein was measured by ELISA. Data are expressed as mean ± SE for each group (e1) (^*∗*^ *P* < 0.05 versus control, ^#^ *P* < 0.05 versus LPS group). PGD_2_ protein was also measured by ELISA. Data are expressed as mean ± SE for each group (e2) (^*∗*^ *P* < 0.05 versus control). ((b1), (b2), (d1), (d2), (f1), (f2)) We also dulicated our test in primary rat lung fibroblasts ((b1), (d1)) (^*∗*^ *P* < 0.05 versus control, ^#^ *P* < 0.05 versus LPS group). ((b2), (d2)) No significant change in the protein expression of COX-1 was observed after treatment with LPS in primary rat lung fibroblasts. (^*∗*^ *P* < 0.05 versus control, ^#^ *P* < 0.05 versus LPS group, (f1)). (^*∗*^ *P* < 0.05 versus control, (f2)). All experiments were repeated in triplicate.

To determine whether exogenous resolvin D1 modulates COXs (COX-1 and COX-2) expression after LPS stimulation, we reassessed COXs (COX-1 and COX-2) protein at 6 hours with various concentrations (10, 50, or 100 nM) of resolvin D1 treatment in HFL-1 cell line; we observed that resolvin D1 inhibited COX-2 protein expression in a dose-dependent manner in HFL-1 cell line (Figure 2(a1, c1)). Thus, using 100 nM resolvin D1 treatment in primary rat lung fibroblasts, we also observed that resolvin D1 inhibited COX-2 protein expression (Figure 2(b1, d1)). However, no significant change in the protein expression of COX-1 was observed after treatment with LPS or/and resolvin D1 both in HFL-1 cell line and primary rat lung fibroblasts (Figure 2(a2, b2, c2, d2)). Moreover, after cells were incubated with resolvin D1 for 6 hours, PGE_2_ and PGD_2_ protein levels in the supernatant were measured by ELISA (Figure 2(e1, e2, f1, f2)). PGE_2_ secretion in HFL-1 cell line was inhibited by resolvin D1 in a dose-dependent manner, decreasing from 407.8 ± 28.53 pg/mL in 0 nM resolving-D1-treated cells to 327.9 ± 18.12 pg/mL in 10 nM resolvin-D1-treated fibroblasts and then further still to 253.7 ± 28.29 pg/mL in 100 nM resolvin-D1-treated fibroblasts (*P* < 0.05). PGE_2_ secretion in primary rat lung fibroblasts was inhibited by 100 nM resolvin D1 as well (*P* < 0.05). In contrast, although PGD_2_ levels increased with LPS treatment, no significant change in the PGD_2_ production was observed between LPS group and resolvin D1 plus LPS groups both in HFL-1 cell line and primary rat lung fibroblasts (Figure 2(e2, f2)).

### 3.3. The Effect of Resolvin D1 on Expression of COXs (COX-1 and COX-2) and PGE_**2**_ and PGD_**2**_ Production at 48 Hours in HFL-1 Cell Line and Primary Rat Lung Fibroblasts Stimulated with LPS

**Figure 3:**
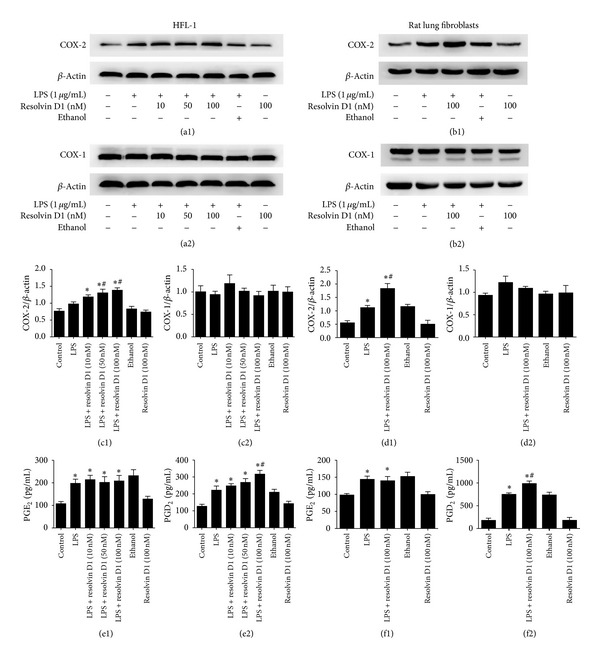
The Effect of resolvin D1 on expression of COXs (COX-1 and COX-2) and PGE_2_ and PGD_2_ production at 48 hours in lung fibroblasts stimulated with LPS. ((a1), (a2), (c1), (c2)) HFL-1 cells were incubated with LPS after 48 hours in the presence or absence of 10, 50, or 100 nM of resolvin D1. COX-2 protein was detected by western blot (^*∗*^ *P* < 0.05 versus control, ^#^ *P* < 0.05 versus LPS, LPS + resolvin D1 (10 nM) groups). No significant change in the protein expression of COX-1 was observed after treatment with LPS in HFL-1 cells. ((e1), (e2)) Supernatants from HFL-1 cells treated with resolvin D1 at 0, 10, 50, or 100 nM in the presence of LPS (1 *μ*g/mL) for 48 hours were collected and PGE_2_ protein was measured by ELISA. Data are expressed as mean ± SE for each group (e1) (^*∗*^ *P* < 0.05 versus control). PGD_2_ protein was measured by ELISA. Data are expressed as mean ± SE for each group (e2) (^*∗*^ *P* < 0.05 versus control, ^#^ *P* < 0.05 versus LPS group). ((b1), (b2), (d1), (d2), (f1), (f2)) We also dulicated our test in primary rat lung fibroblasts. (d1) (^*∗*^ *P* < 0.05 versus control, ^#^ *P* < 0.05 versus LPS group). (d2) There was no significant change in COX-1 expression after LPS treatment in primary rat lung fibroblasts. (^*∗*^ *P* < 0.05 versus control, (f1)). (^*∗*^ *P* < 0.05 versus control, ^#^ *P* < 0.05 versus LPS group, (f2)). All experiments were repeated in triplicate.

To determine whether treatment with exogenous resolvin D1 affected the secondary increase of COX-2 expression after LPS stimulation, we also reassessed COXs (COX-1 and COX-2) protein at 48 hours after various concentrations (10, 50, or 100 nM) of resolvin D1 treatment in HFL-1 cell line, we observed that resolvin D1 promoted COX-2 protein expression in a dose-dependent manner in HFL-1 cell line (Figure 3(a1, c1)). Thus, Using 100 nM resolvin D1 treatment in primary rat lung fibroblasts, we also observed that resolvin D1 increased COX-2 protein expression (Figure 3(b1, d1)). However, no significant change in the protein expression of COX-1 was observed after treatment with LPS or/and resolvin D1 both in HFL-1 cell line and primary rat lung fibroblasts (Figure 3(a2, b2, c2, d2)). We also measured secretion of PGE_2_ and PGD_2_ production following LPS or/and resolvin D1 treatments. Interestingly, in contrast to our result at 6 hours, although levels of PGE_2_ increased after LPS treatment, no significant change in the PGE_2_ production was observed between LPS group and resolvin D1 plus LPS groups both in HFL-1 cell line and primary rat lung fibroblasts (Figure 3(e1, f1)). Furthermore, PGD_2_ secretion was promoted by resolvin D1 in a dose-dependent manner in HFL-1 cell line, increasing from 222.6 ± 25.11 pg/mL in 0 nM resolvin-D1-treated cells to 248.6 ± 14.6 pg/mL following 10 nM resolvin-D1-treated fibroblasts and 316.8 ± 25.33 pg/mL in 100 nM resolvin D1-treated fibroblasts (*P* < 0.05) (Figure 3(e2)). PGD_2_ secretion in primary rat lung fibroblasts was promoted by 100 nM resolving- D1 as well (*P* < 0.05) (Figure 3 (f2)).

### 3.4. COX-2 Protein Expression is Partly through the Activation of ERK1/2 and PI3K/AKT Signaling Pathways in Primary Rat Lung Fibroblasts Stimulated with LPS

**Figure 4:**
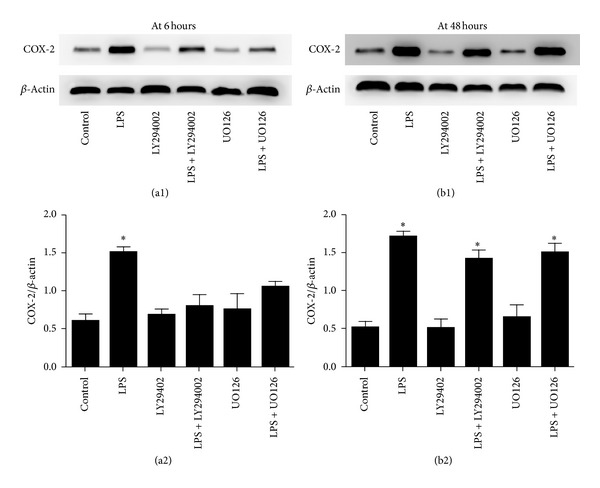
COX-2 protein expression is partly through the activation of ERK1/2 and PI3K/AKT signaling pathways in primary rat lung fibroblasts stimulated with LPS. ((a1), (a2)) COX-2 protein expression induced by LPS in primary rat lung fibroblasts at 6 hours (the first COX-2 expression peak) was suppressed by inhibitors of PI3K/AKT (LY294002) and ERK1/2 (UO126) (^*∗*^ *P* < 0.05 versus control, LPS + LY294002, LPS + UO126, LY294002, and UO126 groups). There is no significant change between control, LY294002, and UO126 groups (*P* > 0.05). ((b1), (b2)) COX-2 protein expression induced by LPS in primary rat lung fibroblasts at 48 hours (the second COX-2 expression peak) was not suppressed by inhibitors of PI3K/AKT (LY294002) and ERK1/2 (UO126) (^*∗*^ *P* < 0.05 versus control, LY294002, and UO126 groups). There is no significant change between LPS, LPS + LY294002 and LPS + UO126 groups (*P* > 0.05). All experiments were repeated in triplicate.

Lipopolysaccharide (LPS) activates intracellular signalling pathways of a remarkable complexity [29], including activation of PI3K/AKT and ERK1/2 signaling pathways in fibroblasts [30]. To determine whether LPS-induced COX-2 expression in primary rat lung fibroblasts is through the activation of ERK1/2 and PI3K/AKT signaling pathways, we assessed the effects of specific inhibitors of ERK1/2 and PI3K/AKT on COX-2 protein expression. We found that COX-2 protein expression induced by LPS in primary rat lung fibroblasts at 6 hours (the first COX-2 expression peak) was suppressed by inhibitors of PI3K/AKT (LY294002) and ERK1/2 (UO126) (^*∗*^ *P* < 0.05 versus control, LPS + LY294002, LPS + UO126, LY294002, and UO126 groups). There is no significant change between control, LY294002 and UO126 groups (*P* > 0.05) (Figure 4(a1, b1)), whereas COX-2 protein expression induced by LPS in primary rat lung fibroblasts at 48 hours (the second COX-2 expression peak) was not suppressed by inhibitors of PI3K/AKT (LY294002) and ERK1/2 (UO126) (^*∗*^ *P* < 0.05 versus control, LY294002 and UO126 groups). There is no significant change between LPS, LPS + LY294002, and LPS + UO126 groups (*P* > 0.05) (Figure 4(a2, b2)).

### 3.5. Resolvin D1 Suppressed Phosphorylation of PI3K/AKT and ERK2 in Primary Rat Lung Fibroblasts Stimulated with LPS

**Figure 5:**
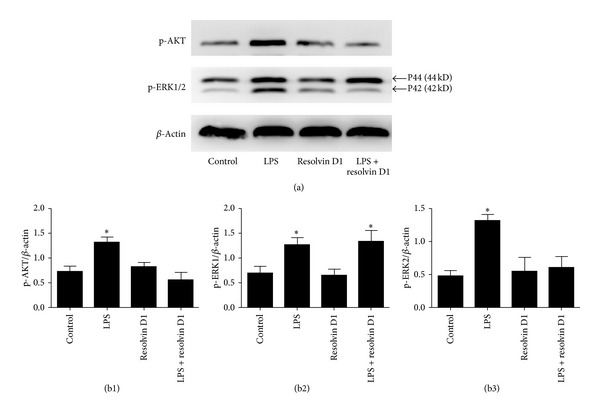
Resolvin D1 suppressed phosphorylation of PI3K/Akt in primary rat lung fibroblasts stimulated with LPS. Western blot analysis of P-AKT and phosphorylated ERK1/2. **β**-actin served as a loading control. Cultured and serum-deprived primary rat lung fibroblasts were treated with lipopolysaccharide (LPS) for 30 minutes for P-AKT and phosphorylated ERK1/2 measurement, with or without preincubation with resolvin D1. ((a), (b1), (b3)) The expressions of p-AKT and the phosphorylated ERK2 (P42) were stimulated by LPS for 30 minutes in primary rat lung fibroblasts, significantly downregulated by resolvin D1 (^*∗*^ *P* < 0.05 versus control, LPS + resolvin D1 groups). ((a), (b2)) However, no significant change in the expression of the phosphorylated ERK1 (P44) was observed between LPS group and LPS plus resolvin D1 group. Results shown are representative of four independent experiments.

To investigate the activity of the PI3K/AKT and ERK1/2 signal pathways in primary rat lung fibroblasts stimulated by LPS after treatment with resolvin D1, we detected p-AKT, a downstream molecule of PI3K, and the phosphorylation of ERK1/2. In a pilot studies, we performed the time course of LPS-induced p-AKT and the phosphorylated ERK1/2 from 0 to 120 min. The peaked expressions of p-AKT and the phosphorylated ERK1/2 were found at 30 min after LPS stimulation, similar to results reported previously [31]. As indicated in Figure 5(a, b1, b2, b3), the expressions of p-AKT and the phosphorylated ERK2 (P42) were stimulated by LPS at 30 minutes in primary rat lung fibroblasts, significantly downregulated by resolvin D1 (^*∗*^ *P* < 0.05) versus control, LPS + resolvin D1 groups. However, no significant change in the expression of the phosphorylated ERK1 (P44) was observed between LPS group and LPS plus resolvin D1 group.

## 4. Discussion

 This study confirms that COX-2 protein expression peaks initially at 6 hours but then also at 48 hours after LPS stimulation in lung fibroblasts, while no significant change in the protein expression of cyclooxygenase-1 was observed after treatment with LPS in lung fibroblasts. Moreover, PGE_2_ levels were peaked only at 6 hours and PGD_2_ levels increased and peaked at 48 hours in lung fibroblasts after LPSchallenge. In our model of LPS-induced acute inflammation, treatment with resolvin D1 reduced COX-2 protein expression and PGE_2_ production at 6 hours, whereas treatment with resolvin D1 promoted COX-2 protein expression and PGD_2_ production at 48 hours. Finally, we also demonstrated that resolvin D1 inhibited COX-2 expression at 6 hours, which is partly through PI3K/AkT and ERK2 signalling pathways.

 Evidence has emerged indicating that resolution of acute lung inflammation and injury is an active process [3, 8]. And edema fluid must be cleared for patients with ALI/ARDS, which is a key step in the resolution of ALI/ARDS [32]. We previously reported that intravenous *β*-agonists (salbutamol) reduced extravascular lung water and improved lung injury in ARDS patients [33]. However, in a multicentre, randomised controlled clinical trial, we found that intravenous infusion of salbutamol given to patients with early ARDS significantly increased 28-day mortality, and treatment was poorly tolerated because of tachycardia, arrhythmias, and lactic acidosis [34]. Therefore, identification of new molecules that promote resolution of acute lung inflammation and injury with fewer side effects is important. Resolvin D1, as one of the endogenous lipid mediators generated during the resolution phase of acute inflammation from omega-3 fatty acid, exhibits potent anti-inflammatory and proresolution actions in different models of inflammation [17–25, 35]. Of interest, transgenic expression of an omega-3 fatty acid mice is protected from colitis in a model of gastrointestinal inflammation [36], with significantly increasing levels of resolvins [36]. Moreover, enteric feeding of supplements enriched with omega-3 fatty acids improves clinical outcomes in ARDS [37]. Thus, it is likely that resolvins would also promote the resolution of airway injury and inflammation.

 Recent research has suggested that resolvin D1 attenuated lung inflammation of LPS-induced acute lung injury [25]. Resolvin D1 markedly reduced the expression of cyclooxygenase-2 (COX-2) in acute lung injury induced by LPS [25]. Resolvin D1 inhibited interstitial fibrosis in the obstructed kidney via inhibition of local fibroblast proliferation [38]. In carrageenin-induced pleurisy in rats, COX-2 protein expression peaked initially at 2 hours, and at 48 hours, there was a second increase in COX-2 expression in inflammatory cells separated from the inflammatory exudates [39]. Our data clearly demonstrated that the expression of COX-2 protein was increased and peaked initially 6 hours after LPS stimulation in lung fibroblasts. This was also associated with maximal PGE_2_ synthesis. However, following 48 hours after LPS stimulation, there was a second increase in COX-2 expression, this time associated with maximal PGD_2_ synthesis. Thus, as inflammation progresses into resolution, PGE_2_ synthesis declines, giving way to a prominence of COX-2-derived PGD_2_, both of which play important roles in mediating resolution of inflammation. This data indicates that COX-2 may be proinflammatory (via PGE_2_ expression) during the development of inflammation, but anti-inflammatory (via PGD_2_ expression) during resolution in lung fibroblasts. our data also demonstrated that COX-1 protein expression is not stimulated by LPS in lung fibroblasts, being consistent with the notion that COX-1 is a housekeeping enzyme and COX-2 is induced by various inflammatory or infectious stimulations [40]. 

 Recent research has suggested a beneficial role for COX-2-derived PGs, including anti-inflammatory and antifibrotic actions in the resolution of inflammation as well as in the early stages of the inflammatory response [26, 39, 41]. To be a versatile enzyme in the inflammatory response, COX-2 possesses the ability to drive inflammation under certain conditions and to resolve inflammation under other conditions. In a model of spontaneously resolving ALI, selective COX-2 inhibition results in prolonged inflammation, in part, by decreasing production of proresolving mediators [26, 41]. So, resolvins and COX-2-derived prostaglandins of the D_2_ and J_2_ series, as an increasingly important family of immunoregulatory lipid mediators, were crucial to the timely recovery from ALI [26, 42].

 Our results also demonstrated that the expression of COX-2 as well as PGE_2_ production by lung fibroblasts were significantly inhibited by resolvin D1 after 6 hours of LPS treatment suggesting that resolvin D1 has a potential anti-inflammatory role in lung fibroblasts during the onset of inflammation. Therefore, the data are consistent with the previous findings that resolvin D1 reduces the expression of cyclooxygenase-2 (COX-2) in acute lung injury induced by LPS [25]. Of interest, the expression of COX-2 by lung fibroblasts of its second increase (48 hours) was significantly promoted by resolvin D1. In addition, consistent with the results above, resolvin D1 promoted the production of PGD_2_ in the supernatants. Therefore, we identify a novel biphasic role of resolvin D1 on the expression of COX-2 and the production of PGE_2_ and PGD_2_, suggesting that resolvin D1 has a potential anti-inflammatory and proresolving roles in LPS-stimulated lung fibroblasts.

 In the present study, we investigated the molecular mechanisms in which resolvin D1 significantly inhibited the expression of COX-2 in LPS-stimulated lung fibroblast cells at 6 hours and promoted the expression of COX-2 in LPS-stimulated lung fibroblast cells at 48 hours. PI3K/Akt and ERK1/2 signaling pathways have been shown to play important roles in LPS-induced expression of COX-2 in many types of cells [31, 43–45], and we investigated whether LPS-induced the expression of COX-2 in lung fibroblast cells was mediated via PI3K/AKT and ERK1/2 signaling pathways. As expected, we found that LPS induced phosphorylation of PI3-K and ERK1/2 in lung fibroblast cells. The levels of phosphorylation of PI3K and ERK1/2 increased from 10 min and remained elevated until 120 min after LPS stimulation and specially increased significantly at 30 minutes. These findings are consistent with the studies in [33, 43, 44]. We further used inhibitors of PI3K/AKT (LY294002) and ERK1/2 (UO126), which demonstrated that the inhibitors inhibited the effects of LPS on COX-2 expression at 6 hours which is similar to a recent report [46]. However, inhibitors did not inhibit the effects of LPS on the COX-2 expression at 48 hours, and it seems that PI3K/AKT and ERK1/2 signaling pathways serve as negative regulators of the COX-2 expression induced by LPS at 48 hours. In addition, we also demonstrated that resolvin D1 significantly inhibited LPS-induced phosphorylation of PI3K/AKT and ERK2 (Figure 5), implicating the involvement of PI3K/AKT and ERK2 in resolvin-D1-induced inhibitory effects on the expression of COX-2 in LPS-stimulated lung fibroblast cells at 6 hours.

 ERK1 and ERK2 are generally described as homologous molecules with parallel activities, kinetics, and substrates [47]. The proteins are 44 and 42 kDa, respectively, and share 85% sequence homology [47]. The fact that these proteins have structural differences, however, raises the possibility that they may have different functions as well [47]. For example, ERK1 and ERK2 are thought to have different roles in Ras-mediated transformation, in proliferation, and so on [47, 48]. Our results also showed that resolvin D1 suppressed LPS-induced phosphorylation of ERK-2, but not ERK-1. 

## 5. Conclusions

This study has demonstrated that the COX-2 protein expression peaks initially at 6 hours but then also at 48 hours after LPS stimulation in isolated lung fibroblasts. Moreover, resolvin D1 has a novel biphasic role on expression of COX-2 and production of PGE_2_ and PGD_2_, whereby resolvin D1 has an anti-inflammatory and proresolving activity in lung fibroblasts following LPS stimulation. Therefore, our study, part of which was the understanding of anti-inflammatory, proresolution molecules, and their counter-regulatory signalling pathways, may provide a novel target for the design of therapeutic strategies for controlling LPS-induced ALI and highlight a new insight into the role of fibroblasts in ARDS. Future experiments should be required to determine which signaling pathways are involved regarding effect of resolvin D1 on the second COX-2 expression peak, which is currently under investigation.

## Abbreviations

ADRS: Acute respiratory distress syndrome
ALI: Acute lung injury
LPS: Lipopolysaccharide
COX-1: Cyclooxygenase-1
COX-2: Cyclooxygenase-2
PGE_2_: ProstaglandinE_2_
PGD_2_: ProstaglandinD_2_
PGs: Prostaglandins.
